# Genome and Transcriptome Analyses Provide Insight into the Euryhaline Adaptation Mechanism of *Crassostrea gigas*

**DOI:** 10.1371/journal.pone.0058563

**Published:** 2013-03-12

**Authors:** Jie Meng, Qihui Zhu, Linlin Zhang, Chunyan Li, Li Li, Zhicai She, Baoyu Huang, Guofan Zhang

**Affiliations:** 1 Institute of Oceanology, Chinese Academy of Sciences, Qingdao, China; 2 University of Chinese Academy of Sciences, Beijing, China; Auburn University, United States of America

## Abstract

**Background:**

The Pacific oyster, *Crassostrea gigas*, has developed special mechanisms to regulate its osmotic balance to adapt to fluctuations of salinities in coastal zones. To understand the oyster’s euryhaline adaptation, we analyzed salt stress effectors metabolism pathways under different salinities (salt 5, 10, 15, 20, 25, 30 and 40 for 7 days) using transcriptome data, physiology experiment and quantitative real-time PCR.

**Results:**

Transcriptome data uncovered 189, 480, 207 and 80 marker genes for monitoring physiology status of oysters and the environment conditions. Three known salt stress effectors (involving ion channels, aquaporins and free amino acids) were examined. The analysis of ion channels and aquaporins indicated that 7 days long-term salt stress inhibited *voltage-gated Na^+^/K^+^ channel* and *aquaporin* but increased *calcium-activated K^+^ channel* and *Ca^2+^ channel*. As the most important category of osmotic stress effector, we analyzed the oyster FAAs metabolism pathways (including taurine, glycine, alanine, beta-alanine, proline and arginine) and explained FAAs functional mechanism for oyster low salinity adaptation. FAAs metabolism key enzyme genes displayed expression differentiation in low salinity adapted individuals comparing with control which further indicated that FAAs played important roles for oyster salinity adaptation. A global metabolic pathway analysis (iPath) of oyster expanded genes displayed a co-expansion of FAAs metabolism in *C. gigas* compared with seven other species, suggesting oyster’s powerful ability regarding FAAs metabolism, allowing it to adapt to fluctuating salinities, which may be one important mechanism underlying euryhaline adaption in oyster. Additionally, using transcriptome data analysis, we uncovered salt stress transduction networks in *C. gigas*.

**Conclusions:**

Our results represented oyster salt stress effectors functional mechanisms under salt stress conditions and explained the expansion of FAAs metabolism pathways as the most important effectors for oyster euryhaline adaptation. This study was the first to explain oyster euryhaline adaptation at a genome-wide scale in *C. gigas*.

## Introduction

As a marine invertebrate, the Pacific oyster, *Crassostrea gigas* (Thunberg, 1793) (Biovalvia, Mollusk), shows the highest aquaculture production (4.4 million tons/year) in the world (Food and Agriculture Organization of the United Nations, 2009, http://www.fao.org) and is one of the best-studied mollusks [1]. This species inhabits coastal zones and plays an important part in estuarine ecology [2]. Unlike other intertidal animals, oysters attached to rocks and cannot move to different locations, and they have evolved many regulatory mechanisms to adapt to highly dynamic and stressful environments [3]. The oyster’s tolerance to extreme stress conditions makes it an excellent study organism for examining stress response and adaptation [4].

Salt stress is an important component of intertidal environments [5]. In such areas, the sea water varies from nearly fresh water, when there is rain, to highly saline and dry salt conditions, when there is drying between tidal inundations [6]. Therefore, oysters experience large and sometimes rapid fluctuations in salinity and thrive within a wide range of optimal salinities, from below salt 10 to in excess of salt 35 [7]. Low salinity is important for the survival and distribution of marine animals [8]. Most marine invertebrates are demonstrated to suffer large-scale mortalities when the salinity dropped [9], [10]. Intertidal areas are subject to frequent low salinity during summer [7], which is one important reason for oysters large scale mortalities. Facing salinity stress, osmoconforming oysters exhibit a number of functional mechanisms to prevent their internal medium from fluctuating widely. Their physiological reactions and tolerance to changes in salinities have been studied by numerous authors [5], [11], [12], [13], [14]. The salt stress responses have been shown to encompass several aspects, including reversible changes in protein and RNA synthesis, alteration of the patterns of multiple molecular forms of different enzymes, and regulation of ionic content and cell volume [15]. In oyster, salt stress mainly induces an osmotic response. Many studies have revealed that intracellular free amino acids (FAAs) predominantly contribute to intracellular osmolality and to cell volume regulation in oysters [11]. The functions of FAAs as osmolytes have been reported by previous studies [16]. These reports have mainly focused on changes in enzymes activities, but the related regulatory mechanism has been nearly ignored and is still poorly understood [15].

The availability of the *C. gigas* genome sequences enabled us to study oyster salt stress responses at a whole-genome scale [17]. iPath (http://pathways.embl.de) analysis is one useful tool for visualizing and analyzing stress metabolic pathways using genome information [18], [19]. KEGG orthologous group identifiers (KOs) can be used to map to iPath to investigate their metabolism relationships. Additionally, transcriptome sequencing enabled us to monitor mRNA expression changes of salt stress effectors through pathway analysis in marine bivalves, including oysters [20], [21], [22]. In previous studies, the transcriptome of the Pacific oyster, *C. gigas*, subjected to 8h short-term low salinity stress (salt 8) was analyzed [23]. However, these authors did not focus on salt stress effectors, or FAAs metabolism in particular. Additionally, they did not use oyster genome data to annotate salt-responsive genes.

In our experiments, we analyzed the functional mechanism of salt stress effectors (especially FAA metabolism) involved in 7 days long-term stress responsiveness. The final aim of our experiment was to explain oyster euryhaline adaptation using physiology experiment, transcriptome data and quantitative real-time PCR (Q-RT PCR) verification. To obtain a better understanding of gene co-regulatory networks in oysters, we conducted a number of further analyses of the salt stress effectors, especially FAAs metabolism, under salt stress conditions. First, we conducted physiological experiments to monitor the changes in FAAs contents under salt stress conditions. Second, we focused on FAAs metabolism pathways analysis and uncovered the FAAs functional mechanisms under salt stress conditions. The expression differentiations of FAAs metabolism key enzyme genes were also investigated in low salinity adapted individuals compared with control. Third, we compared osmotic effector genes at the genome-wide scale using five euryhaline species (including *C. gigas*) and three stenohaline species. Compared with seven other species, oyster expanded KOs were used for iPath analysis which revealed their co-expansion in FAAs metabolism pathways. The genome-wide pathway comparison was the first such analysis to be employed in studies in oysters. Our results partially explained the mechanism of oyster salt stress adaptation and increased the understanding of oyster salt stress regulation.

## Materials and Methods

### Ethics statement

The oysters used in the present study were marine-cultured animals, and all of the experiments were conducted according to the regulations of the local and central government.

### Oyster collection and experimental treatment

#### Oyster collection

Oysters for the quantitative real-time PCR (Q-RT-PCR) experiments and FAAs contents measurements were treated under the same condition with transcriptome sequencing [17]. All the oysters were purchased from the Yan Dunjiao aquaculture farm in Weihai, Shandong Province, China and acclimatized in seawater tanks for 1 week before processing. A total of 42 oysters were distributed among seven groups in separate tanks for 7 days long term salt stress treatment. The mortality rates were monitored daily and the gills were collected for further RNA extractions and FAAs contents measurements.

#### Salinity treatment

Sea salt, sea water and aerated fresh water were used to prepare the experimental water to produce the following gradient: salt 5, 10, 15, 20, 25, 30, and 40. The salinities were measured by a Knauer semimicro osmometer. Salt 5, 10, 15, 20 and 25 were hypo-osmotic stress for oysters, while salt 40 was the hyper-osmotic stress. Salt 30 was the normal salinity.

#### Oysters larvae hypo-osmotic stress acclimation

The eyed larvae stage oysters were equally been divided into two groups in two well-aerated aquaria (80 L) during acclimation. One group was kept in normal salinity water (salt 30) and used as control. The other group was subjected to low salinity acclimation (salt 15) for 8 days. After 8 days larvae low salinity acclimation, the survival individuals were selected as adapted ones. During the acclimation, the salinity was measured each day just before the seawater exchange. After 8 days treatment, the oysters were transferred into the normal salinity condition (salt 30). Then all oysters were cultured in the same sea area for seven months. Fifteen oysters were collected in each group and the gills were immediately frozen for further RNA extraction.

### RNA extraction

For Q-RT PCR verification, the gills of oysters were collected. Total RNA was isolated using the TRIzol reagent (Invitrogen). The RNA yield and purity were determined spectrophotometrically (BioPhotometer, Eppendorf, Hamburg, Germany) at A260 and A280. The RNA integrity was assessed via electrophoresis in a 1.2% agarose gel. Each RNA sample was used for gene expression detection separately.

### Measurement of FAAs contents

To homogenize oyster gills, 10 volumes (W/V) of 80% ethanol were used. Then, 0.3 g samples were centrifuged at 10,000×g for 15 min, and the supernatants were dried and analyzed to determine the FAAs contents. The amounts of FAAs in the gills were determined via high-performance liquid chromatography (HPLC) in a Cosmosil 5C18AR-2 packed column (250 mm×4.6 mm i.d., particle size 5 µm, Nacalai Tesque, Kyoto, Japan) following the method of Sato *et al.* [24], [25]. The contents of alanine (Ala), arginine (Arg), glycine [25], proline (Pro) and taurine (Tau) were determined.

### Salt stress responsive genes identification

Transcriptome data were downloaded from the NCBI database, under the GEO number GSE31012 [17]. We also obtained the differentially expressed genes (DEGs) and their Gene Ontology (GO) annotations in salinity stress group (salt5, 10, 15, 20, 25 and 40) compared with normal salinity condition (salt30) from the published data [17] We further conducted the Kyoto Encyclopedia of Genes and Genomes (KEGG) (http://www.genome.jp/kegg/) annotations of DEGs and the significantly enriched pathways were identified via a chi-square test (*P*<0.05).

### Search for marker genes

In our previous studies, the salt stress treatments were divided into four groups: extreme low salinity (salt 5), low salinity (salt 10 and 15), moderate low salinity (salt 20 and 25), and moderate high salinity (salt 40) [17]. To search for marker genes that reflected the special salinity stress conditions, we investigated the DEGs showing a fold change of more/less than 10 between controls and the four groups (genes with RPKM <1 were removed). The obtained genes were then used to draw Venn diagrams at the following website:http://bioinformatics.psb.ugent.be/ webtools/Venn/.

### Charting metabolic pathways (Ipath) for expanding genes

KEGG Orthology (KO) annotations of the genes were performed using InterProScan software in the following species genome database: the intertidal animals (*C. gigas*, *Capitella capitata*, *Lottia gigantean*, *Strongylocentrotus purpuratus* and *Helobdella robusta*) and the stenohaline terrestrial animals (*Drosophila melanogaster*, *Apis mellifera* and *Homo sapiens*) (Table S1). The obtained KOs were mapped to KEGG database to investigated their metabolic maps. We compared the KOs numbers among eight species. The expanded and contracted KOs and maps comparing the intertidal animals vs. stenohaline terrestrial animals and oysters vs. all the other seven species were identified via a chi-square test (*P*<0.05). For example, we identified 118 KOs counts in map00350 for the oyster and the mean of 80 KOs counts for the other seven species displayed in Table S1. The chi-square test statistic and the corresponding *P*-value were calculated as 0.000633. iPath, provided powerful tools to visualize, navigate, explore and analyze all, or a subset of, various pathway maps. The expanded KOs comparing intertidal animals vs. stenohaline terrestrial animals and oysters vs. all the other animals (*P*<0.05) were also mapped onto the overview metabolic network iPath to observe their functional relationships (http://pathways.embl.de/iPath2.cgi) [19].

### Quantitative real-time PCR validation

For the quantitative real time PCR (Q-RT-PCR) analysis, the RNA sample was reverse transcribed using a cDNA synthesis kit (Takara, DRR420), and Q-RT-PCR was performed with the ABI7500 fast Real-Time Detection System (Applied Biosystems, USA). Primers for seventeen FAAs metabolism-related genes and two stress marker genes (Table S2) were used to amplify PCR products. The elongation factor (EF) gene was chosen as a reference gene for internal standardization. The Q-RT-PCR amplifications were carried out in triplicate in a total volume of 20 µl containing 10 µl of SYBR Green 2× Supermix (Takara), 1 µl of 1∶100 diluted cDNA, 0.4 µl each of the forward and reverse primers, 0.4 µl of ROX Dye II, and 7.8 µl of DEPC H_2_O. The PCR program involved two steps: 95 °C for 30 s, followed by 40 cycles at 95 °C for 3 s and 60 °C for 30 s. The analysis was based on the Ct values of the PCR products. A melting curve analysis of the products was performed at the end of each PCR amplification to confirm that only one PCR product was amplified and detected. The differences in the Ct values between the amplified genes and EF gene (ΔCt) were calculated. The blank group was used as the reference sample (i.e., the calibrator). The ΔCt for each sample was then subtracted from the ΔCt of the calibrator; this difference is called the ΔΔCt value. The expression level of the target genes then was calculated as 2^–ΔΔCt^.

For library validation, these selected genes belong to the ‘Searches for marker genes under the examined salt stress conditions’ and ‘Salt stress effectors-FAAs metabolism during the salt stress response’ categories. In the analysis of their expression, the fold changes (-△△Ct) measured via Q-RT-PCR and determined in the transcriptome data were highly consistent, thereby validating the transcriptome data.

## Results and Discussion

### Physiological changes - FAAs contents of the oyster in response to salt stress

FAAs are the primary osmolytes in marine mollusks, whose osmo-conforming process is vital for environmental salinity adaptation because of a lack of osmotic homeostasis [13], [15]. The results shown in Table 1 indicated that the observed changes in the total FAAs contents were dose-dependent with respect to the osmolality changes in the gills. Compared with the normal salinity (salt 30), the total FAAs contents decreased under hypo-osmolality (salt 5, 10, 15, 20, 25) and reached their lowest level in the salt 10 treatment. It has been speculated that this decrease leads to the permeation of the ambient seawater and increases the water content in the gills [26], [27]. Salt 5 was special which was identified with previous principal component analysis (PCA) of transcriptomes and further indicated that it may exceed the range of oyster salinity tolerance [17]. Under exposure to hyper-osmolality (salt 40), the total FAAs accumulated and increased gill osmolality, allowing the oysters to adapt to 7 days long-term stress. The results shown in Table 1 also suggested that Tau, as the most abundant FAAs component in oysters, contributes predominantly to osmotic regulation. Gly, Ala and Pro played more important roles under hyper-osmolality (salt 40) stress than in hypo-osmolality (salt 10, 15, 20, 25) conditions. The changes in the FAAs contents were the good indications that oysters were actively mounting stress responses during the applied treatments.

**Table 1:** FAAs contents in gills of oysters after 7 days different salinities treatments.

Name/salt	5	10	15	20	25	30	40
Glutamate	0.25	0.15	0.30	0.54	0.37	0.36	0.58
Glycine	0.09	0.08	0.09	0.09	0.13	0.16	0.48
Alanine	0.26	0.09	0.13	0.14	0.13	0.13	0.48
Arginine	0.20	0.21	0.24	0.24	0.26	0.26	0.29
Proline	0.06	0.01	0.01	0.01	0.01	0.04	0.22
Taurine	5.23	3.92	5.19	6.19	7.40	7.86	9.18
Total	6.09	4.62	6.00	7.21	8.30	8.81	11.23

Values of FAAs contents are in gram/kgram dry weight. The content values represent the mean value in three different individuals. Salt 5, 10, 15, 20 and 25 were hypo-osmotic stress, salt 40 was hyper-osmotic stress, and salt 30 was the normal salinity.

### Search for marker genes under the examined salt stress conditions

**Figure 1:**
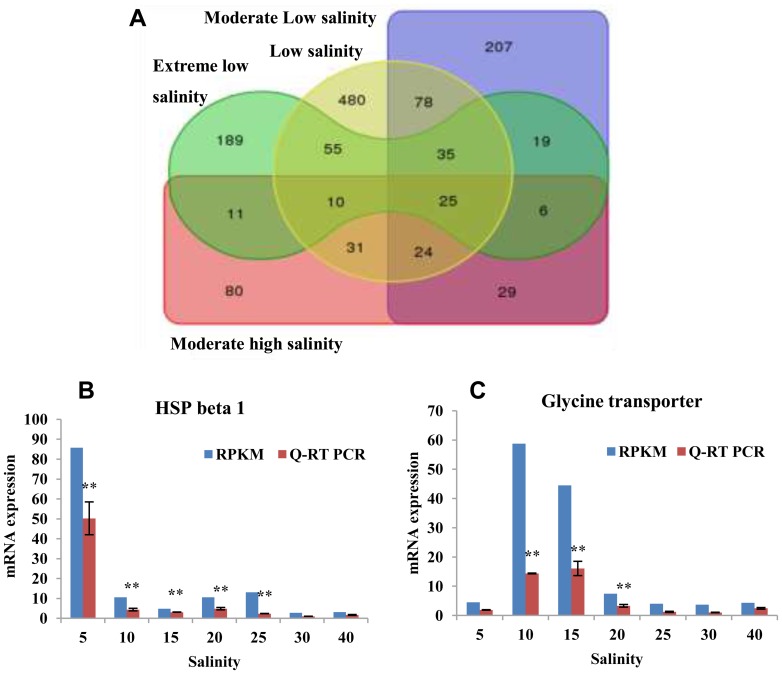
Marker genes search under different salinity treatments. (A) Venn diagram of gene sets identified under various salt stress conditions. The green region indicates ‘extreme low salt stress’ (salt 5); The yellow indicates ‘low salt stress’ (salt 10 and 15); The blue indicates ‘moderate low salt stress’ (salt 20 and 25); The red indicates ‘moderate high salt stress’ (salt 40). **(**B, C) The expressions of *HSP beta 1* (B) and *Glycine transporter* (C) in transcriptome data and Q-RT PCR. For Q-RT PCR data, vertical bars represent the mean ± S.D (N  =  3). Significant differences between salt stress treatment (salt 5, 10, 15, 20, 25 and 40) and control (salt 30) are analyzed by one-way ANOVA (**P<0.01, *P<0.05).

Stress marker genes are used for an increasing extent in eco-toxicology studies, with the aim of detecting the effects of environmental conditions [28]. It is clear that significant mRNA expression changes in molecular marker genes reflect the reaction of cells to a stimulus and may be considered sensitive indicators of the physiological status of oysters and environmental pollutions [29]. Transcriptome data uncovered the stress marker genes in transcriptional level in the genome wide scale. Based on previous studies, the salinity stress treatments were divided into four groups: extreme low salinity (salt 5), low salinity (salt 10, 15), moderate low salinity (salt 20, 25) and moderate high salinity (salt 40) [17]. From the obtained Venn diagram (Fig. 1A), we can see that there were 189, 480, 207 and 80 genes expressed specifically under the extreme low salinity (salt 5), low salinity (salt 10, 15), moderate low salinity (salt 20, 25) and moderate high salinity (salt 40) conditions. These specifically expressed genes (Table S3) were considered candidate marker genes for monitoring each of the examined salt stress conditions in the oysters. For example, one of these genes, *HSP beta 1*, which belongs to the HSP gene family, showed 30-fold increases under the extreme low salinity (salt 5) (Figure 1B), whereas its expression displayed less than 5-fold changes under the other conditions. This may be a good candidate marker gene for extreme low salinity detection. Another potentially useful marker gene, the *sodium- and chloride-dependent glycine transporter* gene, was highly expressed under low salinity (salt 10, 15) conditions (Figure 1C), providing a potential mechanism for oyster hypo-osmotic adaptation.

In conclusion, we identified a group of genes that responded to specific salinities at the transcriptional level to monitor environmental effects, and the information obtained in this analysis also led to a better understanding of the complex stress responses of oysters.

### Salt stress effectors - Ion channels and Aquaporins

Although oyster blood remains isosmotic with the surrounding fluids during acclimation, they are capable of performing some ionic regulation and water transport under osmotic stress [30]. The ion channels and aquaporins were considered as important salt responsive effectors and the related genes displaying more than two fold changes were analyzed.

#### Ion channels

Under osmotic stress conditions, ion channels mediate the ionic steady state not only for Na^+^ and Cl^-^ but also for K^+^ and Ca^2+^ [31]. In our results (Table S4), two copies of *voltage-gated Na^+^ channel* genes and four copies of *voltage-dependent K^+^ channel* genes decreased under low salt stress conditions and reached the lowest level under salt 10 or 15 condition. However, one copy of *calcium-activated K^+^ channel* gene, one copy of *Ca^2+^ channel* gene and one copy of *Cl^-^ channel* gene increased under low salt stress conditions. Under hyper-osmotic stress condition (salt 40), the same changes of these genes were observed (except CGI_10024636 and CGI_10011873) (Table S4). But all changes did not reach two fold.

Previous studies have indicated that when the cell membrane is depolarized, voltage-gated Na^+^/K^+^ channels are activated and inactivated within milliseconds to maintain osmotic balance [32], [33]. Our results showed that after 7 days long-term low salt stress (salt10 or 15), the expression of *voltage-dependent Na^+^/K^+^ channel* genes (Table S4) decreased, which induced changes in Na^+^/K^+^ currents and osmotic pressure in oyster cells to allow them to adapt to environmental changes. This was accordant with previous studies [34]. Ca^2+^ could regulate membrane activities associated with the control of cell volume during hypotonic stress in mollusks [35], [36]. Based on our results, the increased expression of *Ca^2+^ channels* may alter Ca^2+^ currents and activate calcium-activated K^+^ channels to rectify the K^+^ current.

In conclusion, our results indicated that hypo-osmotic stress (salt 10 or 15) decreased the expression of *voltage-gated Na^+^/K^+^ channel* genes and resulted in membrane depolarization, leading to a new osmotic balance. The increased *Ca^2+^ channel* gene expression altered the Ca^2+^ current, which induced increased activities of calcium-gated K^+^ channels to maintain the ionic balance under hypo-osmotic condition (salt 10 or 15). The similar expression changes of these genes were observed under salt 40 condition which suggested the similar mechanism for oyster long term hyper-osmotic stress adaptation.

#### Aquaporins

In addition to maintaining ionic homeostasis under salt stress conditions, oysters also need establish water homeostasis via aquaporins (AQPs) [15]. The functions of AQPs have been studied in animals, plants and bacteria [37], but little was known about them in oysters. Our data (Table S4) showed that three copies of *AQPs* decreased under hypo-osmotic stress conditions, and reached the lowest levels under salt 10 or 15. The same changes were observed under hyper-osmotic (salt 40) conditions. These results may indicated that 7 days long-term stress induced a reduction of AQPs activities to protect against water currents as well as gill cell swelling and shrinkage. Similar results were found in many previous studies under long-term salt stress [38]. The decreased *AQPs* genes expression may be explained by increased protein phosphorylation mediated by long-term stress [39], [40].

### Salt stress effectors – FAAs metabolism during the salt stress response

Osmotic adaptation is considered to be a major component of the oyster salt stress response. To adapt to changes or fluctuations in the salinity of ambient seawater, osmo-conforming marine animals, including oysters, exhibited mechanisms that allow them to adjust the concentrations of intracellular osmolytes to regulate cell volume. Previous studies have shown that intracellular FAAs are the main contributors to the regulation of intracellular osmolality and cell volume in bivalves [11]. However, FAAs osmotic regulation molecular mechanism has been little studied to our knowledge. Under hypotonic stress, internal tissues in oysters, such as the heart, release free amino acids to the hemolymph or bathing medium as part of the regulatory decrease in volume. The whole animal responds by increasing its metabolic rate and releasing ammonia, rather than amino acids, to the bathing medium. The gill or mantle is thought to be the site for the catabolism of the free amino acids released by the internal tissues during hypotonic stress [41]. On the basis of this understanding, enzymes that catalyze rate-limiting steps in the FAAs metabolism are considered to be the salt-stress-responsive effectors. Previous studies have indicated that glycine, proline, alanine, beta-alanine, arginine, and taurine are the major FAAs in *C. gigas* [13]. We mainly focused on these FAAs metabolism key enzymes under salt stress conditions in oysters. The final aim of our experiment was to reveal the FAAs functional mechanisms for oyster salt stress adaptation.

#### Taurine metabolism

**Figure 2:**
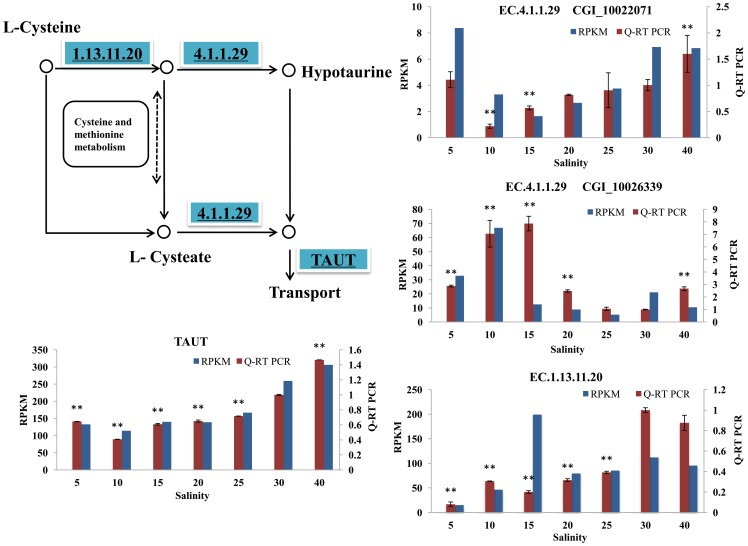
Pathway analysis for taurine metabolism. The map displays selected steps from KEGG map00430, ‘Taurine and hypotaurine metabolism’. A red rectangle indicates increased expression of genes compared with salt 30, while a blue rectangle indicates decreased expression of genes. The EC number with an underline was validated via Q-RT PCR. The transcriptome data and Q-RT PCR data were displayed in the form of columnar diagram. For Q-RT PCR data, vertical bars represent the mean ± S.D (N  =  3). Significant differences between salt stress treatment (salt 5, 10, 15, 20, 25 and 40) and control (salt 30) are analyzed by one-way ANOVA (**P<0.01, *P<0.05).

Taurine, a non-protein amino acid, is a small organic solute that can accumulate to a high intracellular concentration without perturbing macromolecules [42]. Although the functions of taurine are numerous and are still being defined, its osmoregulatory role has been well established [43]. Marine invertebrates, most notably oysters, contain high concentrations of taurine that can be used to regulate tissue osmolarity [27]. Taurine accounts for approximately 80% of the total FAAs content under the condition of hypo-osmolality [44]. Taurine metabolism occurs mainly via two pathways (Fig. 2): through synthesis from cysteine and through taurine uptake facilitated by a high-affinity transport system. In bivalves, the system involved in taurine synthesis from cysteine is well-documented [27], [45]. In this pathway, the sulfhydryl group of cysteine is first oxidized to cysteine sulfinic acid by the enzyme cysteine dioxygenase (EC.1.13.11.20, CDO). Cysteine sulfinic acid, in turn, is decarboxylated by cysteine sulfinic acid decarboxylase (EC.4.1.1.29, CSAD) to form hypo-taurine. It is unclear whether hypo-taurine is then spontaneously or enzymatically oxidized to yield taurine. In our results, the significant decreased *CDO* gene expression under low salt stress conditions may result in the low taurine synthesis rate (confirmed by Q-RT-PCR). However, under high salt stress condition (salt 40), there is no significant change. CSAD (EC.4.1.1.29) represents the rate-limiting enzyme in this metabolic pathway. Two copies of *CSAD* genes showed significantly altered mRNA expressions under salt stress conditions. Interestingly, the expression changes of these two copies displayed differentiation. One copy of *CSAD* gene decreased significantly under low salt stress conditions (salt 10, 15) and increased significantly under high salt stress condition (salt 40). This result was identified based on previous studies and from our experimental physiological data (Table 1), indicating that taurine synthesis would decrease under hypo-osmotic stress and increase under hyper-osmotic stress. However, another copy of *CSAD* gene showed expression differentiation and significantly increased under low salt stress (salt 10, 15, 20) condition. We proposed that this two genes expression differentiation was the sub-functionalization after duplication. In oyster genome research, we found that oyster stress responsive genes are more likely to retain paralogous duplications than all genes on average [17]. The analysis of the relationship between genes differentiation time (Ks) and their expression divergence, we can see that expression divergence exists even in the paralogous with highly similarity sequences (unpublished). *CSAD* may be one typical example of this situation. We further conducted multi-sequence alignment and PFAM domain prediction using the oyster and many other species CSAD protein sequences to studies their genes expressions differentiation mechanisms (Fig. S1). The results showed that the increased gene copy (CGI_10026339) included additional complex regulatory sites in C-terminus of its sequence. For example, there is a zinc finger domain (ZnF_GATA type) in the C-terminus of the protein (Fig. S1). The functions of zinc finger domains are diverse and include DNA recognition, RNA packaging, transcriptional activation, regulation of apoptosis, protein folding and assembly, and lipid binding [46]. Additional zinc finger motifs in CSAD (CGI_10026339) protein may lead to a better understanding of *CSAD* gene broader functions in transcriptional regulation. This hypothesis may be one explanation for their expressions differentiation and need more experimental data. The other mechanism of taurine accumulation is via taurine uptake facilitated by the taurine transporter (TAUT). In the investigated transcriptomes, *TAUT* expression was observed to decrease significantly under low salt stress conditions (salt 5, 10, 15, 20, 25) and increase significantly under high salt stress condition (salt 40), in agreement with other studies and the Q-RT-PCR results. Following previous studies, we can see that the reduction of *TAUT* expression in mollusks under hypo-osmotic conditions was the result of a substantial decrease in the taurine content following prolonged osmotic stress.

In conclusion, the significant down-regulation of the mRNA expression of the *CDO*, *CSAD* and *TAUT* genes led to decreased accumulation of taurine under low salinities. However, the significant increased *CSAD* and *TAUT* genes under hyper-osmotic stress condition induced increased accumulation of taurine. These results were accordant with our physiological data (Table 1).

#### Glycine metabolism

**Figure 3:**
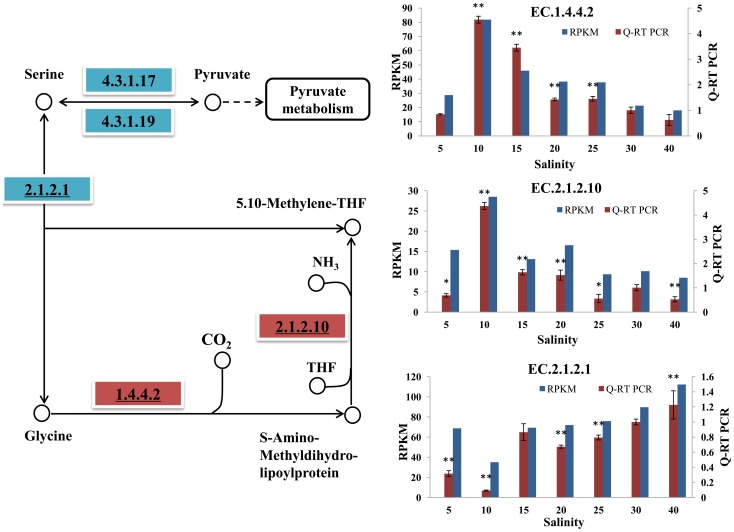
Pathway analysis of glycine metabolism. The map displays selected steps from KEGG map00260, ‘Glycine, serine and threonine metabolism’. A red rectangle indicates increased expression compared with salt 30, and blue indicates decreased expression compared with salt 30. The EC number with an underline was validated via Q-RT PCR. The transcriptome data and Q-RT PCR data were displayed in the form of columnar diagram. For Q-RT PCR data, vertical bars represent the mean ± S.D (N  =  3). Significant differences between salt stress treatment (salt 5, 10, 15, 20, 25 and 40) and control (salt 30) are analyzed by one-way ANOVA (**P<0.01, *P<0.05).

Glycine is degraded mainly via two pathways (Fig. 3). The predominant pathway, which is involved in the glycine cleavage system, leads to the degradation of glycine into ammonia and CO_2_. This process is catalyzed by glycine dehydrogenase (EC.1.4.4.2, GLDC) and aminomethytransferase (EC.2.1.2.10, AMT). The expression profiles determined in both the transcriptome and Q-RT-PCR analyses showed that the genes encoding these two enzymes showed significantly increased expressions under hypo-osmotic stress conditions, reaching the highest expression level under salt 10 condition. Under hyper-osmotic (salt 40) stress condition, *aminomethytransferase* gene significantly decreased and *glycine dehydrogenase* gene did not change significantly. In the second pathway, glycine is degraded through two steps. The first step is the conversion of glycine into serine via serine hydroxymethyl transferase (EC.2.1.2.1, SHMT). This is a reversible reaction and is also the main pathway for glycine biosynthesis from serine. SHMT catalyzes this step with methylene THF tetrahydrofolate (THF), and *SHMT* gene mRNA expression significantly decreased under the hypo-osmotic treatment (salt 10) and significantly increased under hyper-osmotic treatment (salt 40), as confirmed by Q-RT-PCR. These changed enzyme gene expressions indicated that the inter-conversion of glycine and serine may be effected under osmotic stress conditions.

Overall, based on the analysis of key enzyme genes involved in glycine metabolism, we can conclude that oyster increased/decreased glycine degradation mainly through the glycine dehydrogenase and aminomethytransferase pathway under hypo/hyper osmotic stress conditions. The observed expression of these genes supported the finding that glycine content decreased under hypo-osmotic stress and increased under hyper-osmotic stress, in accord with the physiological symptoms seen in our experiments (Table 1).

#### Arginine and proline metabolism

**Figure 4:**
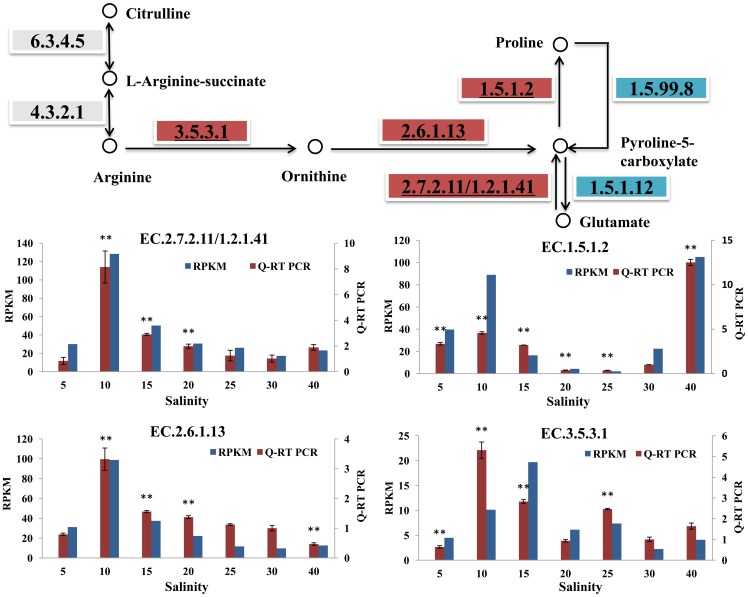
Pathway analysis of arginine and proline metabolism. The map displays selected steps from KEGG map00330, ‘Arginine and proline metabolism’. A red rectangle indicates increased expression genes compared with salt 30, while blue indicates decreased expression of genes, and the gray rectangle indicates no significant changes. The EC number with an underline was validated via Q-RT PCR. The transcriptome data and Q-RT PCR data were displayed in the form of columnar diagram. For Q-RT PCR data, vertical bars represent the mean ± S.D (N  =  3). Significant differences between salt stress treatment (salt 5, 10, 15, 20, 25 and 40) and control (salt 30) are analyzed by one-way ANOVA (**P<0.01, *P<0.05).

Arginine and proline are two important FAAs that play important roles in osmotic stress [13]. We analyzed the expression patterns of key enzyme genes related to the ‘arginine and proline metabolism’ pathway, which involves the co-metabolism of arginine, ornithine, proline and glutamate (Fig. 4).

In animals, intracellular proline levels are mainly controlled via biosynthetic and catabolic pathways [47]. Under osmotic stress condition, proline is synthesized and degraded mainly through the glutamate metabolism pathway [47]. Proline is synthesized via Δ-1-pyrroline-5-carboxylate (Δ-1- P5C) through two successive reductions, which are catalyzed byΔ-P5C synthase (EC 2.7.2.11/1.2.1.41, P5CS) and P5C reductase (EC.1.5.1.2, P5CR). The genes encoding these two enzymes have been identified in oysters and were observed to increase under both hypo- and hyper-osmotic stress conditions (confirmed by Q-RT-PCR). Proline degradation is catalyzed through the sequential action of the mitochondrial enzymes proline dehydrogenase (PDH, EC.1.5.99.8) and P5C dehydrogenase (P5CDH, EC.1.5.1.12). Our data showed that the expressions of the genes encoding these two enzymes decreased under hypo- and hyper-osmotic conditions in a dose-independent manner. At the transcriptional level, the up-regulation of *P5CR* and *P5CS* and down-regulation of *PDH* and *P5CDH* might be correlated with the high accumulation of proline under hypo- and hyper-osmotic stress conditions. The observed proline accumulation under hyper-osmotic stress conditions was in agreement with many previous studies and the data from our physical experiments (Table 1) [13], [41]. This finding may be explained due to the accumulation of proline, as a compatible solute, resulting in an increase in cellular osmolarity that can drive the influx of water or reduce its efflux. However, under hypo-osmotic stress conditions (salt 10, 15), despite the significant increases in the expressions of the proline synthesis-related genes *P5CR* and *P5CS*, the content of proline in oysters did not increase according to the data from our physical experiments (Table 1). This may indicate that proline synthesis is controlled by a complex regulatory mechanism through post-transcriptional processes, as found in previous studies [47]. During stress, genes may be induced either as part of a primary response to minimize damage or as a result of secondary effects. The increase in *P5CR* and *P5CS* expressions may be the result of oxidative stress adaptation. Many other experiments need to be conducted to establish whether these events are a primary response to oxidative stress or if this response is likely to be of adaptive significance.

Arginine, as the other important FAA, played an important role in osmotic stress in the oysters. Arginine is synthesized from citrulline through the sequential action of the cytosolic enzymes argininosuccinate synthetase (EC.6.3.4.5, ASS) and argininosuccinate lyase (EC.4.3.2.1, ASL). The expression of *ASS* and *ASL* did not display significant changes under osmotic stress conditions based on our data. Arginine is degraded via arginase (EC.3.5.3.1) and ornithine aminotransferase (EC. 2.6.1.13). The transcript levels for both of these enzyme genes were significantly increased in response to low salt stress (salt 10 and 15) (confirmed by Q-RT-PCR). These results indicated that arginine synthesis decreased under hypo-osmotic stress conditions, which was in agreement with our physiological data (Table 1). Under hyper-osmotic stress (salt 40), the significant decrease of *ornithine aminotransferase* was also observed by Q-RT PCR. Overall, the gene expression profiles of the investigated key enzymes and physiological experiments (Table 1) indicated that proline accumulated and played an important role in hyper, hypo-osmotic stress conditions. Additionally, the key genes related to the arginine metabolism pathway were significantly induced under osmotic stress. However, the regulatory mechanisms involved in both proline and arginine metabolisms are complex and require more experimental data.

#### Alanine and Beta-alanine metabolism

**Figure 5:**
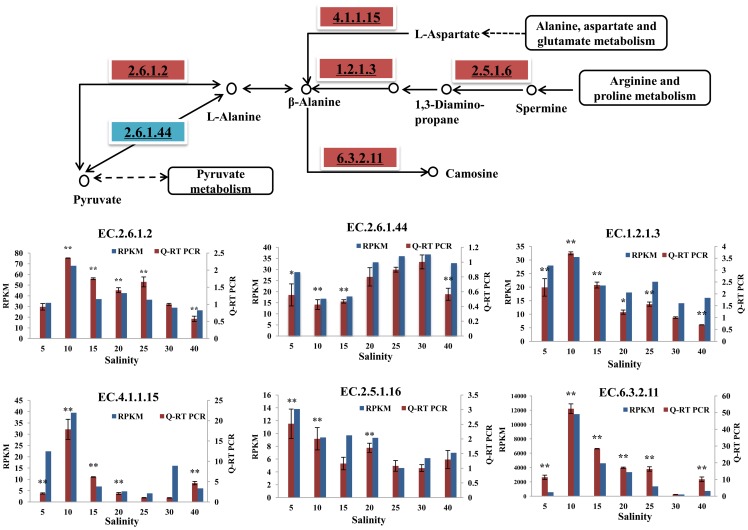
Pathway analysis for alanine and beta-alanine metabolism. The map displays selected steps from KEGG pathways map00473, ‘D-Alanine metabolism’, and map00410, ‘beta-Alanine metabolism’. A red rectangle indicates increased expression of genes compared with salt 30, while blue indicates decreased expression of genes. The EC number with an underline was validated via Q-RT PCR. The transcriptome data and Q-RT PCR data were displayed in the form of columnar diagram. For Q-RT PCR data, vertical bars represent the mean ± S.D (N  =  3). Significant differences between salt stress treatment (salt 5, 10, 15, 20, 25 and 40) and control (salt 30) are analyzed by one-way ANOVA (**P<0.01, *P<0.05).

Cell volume regulation is accomplished by utilizing intracellular free alanine as an osmotic solute in mollusks [11]. In animals, alanine is most commonly produced through the reductive amination of pyruvate via alanine transaminase (EC.2.6.1.2, ALT) following the interconversion of alpha-ketoglutarate (2-oxoglutarate) and glutamate [48] (Fig. 5). Our results showed that two copies of the *alanine transaminase* genes exhibited significant increases under the salt 10 condition and significant decreases under salt 40, indicating that salt stress might strongly affect the interconversion of pyruvate and alanine. Another route for the production of alanine is through the enzyme alanine-glyoxylate transaminase (EC.2.6.1.44, AGT). This reaction involves the interconversion of alanine and pyruvate, coupled with the interconversion of glyoxylate and glycine [49]. However, *AGT* expression decreased significantly under hyper- (salt40) and hypo-osmotic conditions (salt 10 and 15), indicating that this pathway may be inhibited by osmotic stress.

In oysters, beta-alanine, which was found to be enriched through KEGG analysis, plays crucial roles under osmotic stress conditions [11], [13]. There are two major pathways for the synthesis of beta-alanine (Fig. 5). One simple route, involving a single decarboxylation step from aspartic acid, has been found in oysters. ‘Glutamate decarboxylase’ (EC.4.1.1.15, GAD), which catalyzes aspartic acid decarboxylation to produce beta-alanine and CO_2_, showed significant increased mRNA expression levels under hyper- and hypo-osmotic conditions (verified by Q-RT-PCR). The second major pathway involves synthesis from spermine, which is derived from the metabolism of arginine and proline. Within this pathway, two major enzyme genes, the *spermidine synthase* (EC.2.5.1.16, SPDS) and *aldehyde dehydrogenase* (EC.1.2.1.3, ALDH) genes were significantly increased under hypo-osmotic stress conditions. Under hyper-osmotic stress conditions, *aldehyde dehydrogenase* significantly decreased, while *spermidine synthase* did not display significant changes. The degradation of beta-alanine is catalyzed by the ‘ATP-grasp domain-containing protein’ (EC.6.3.2.11, ATPGD1), and genes homologous to those encoding this protein have been found in oysters. The expressions of these genes increased significantly under hypo-osmotic stress, reaching the highest level under the salt 10 condition. Therefore, our data indicated that the oysters showed an increased rate of beta-alanine metabolism to maintain osmotic equilibrium under hypo-osmotic stress conditions. The *ATP-grasp domain-containing protein* also increased significantly under hyper-osmotic stress condition. This may be explained that this gene also participate in many other metabolism processes under stress condition.

Overall, under osmotic stress conditions, the oysters exhibited an increased rate of alanine and pyruvate interconversion via the alanine transaminase pathway. Both the synthesis and degradation of beta-alanine increased under hypo-osmotic stress conditions in oysters, indicating that the turnover rate of beta-alanine was affected at the transcriptional level.

#### FAAs metabolic key enzyme genes expression verification

In order to further validate the important roles of FAAs metabolism for oyster salt stress adaptation, we conducted genes expressions comparisons between low salinity adapted individuals and control. For each group, fifteen individuals were respectively selected to investigate their seventeen FAAs metabolism key enzyme genes expressions using Q-RT PCR.

Gene expression level is a quantitative phenotype that is directly linked to genetic variation [50]. It can be used to investigate the different genetic basis in two different salinity adapted groups. Figure S2 showed most of the investigated key enzyme genes involved in FAAs metabolism displayed significant higher expression levels in low salinity adapted individuals, especially the genes coding for Δ-1-pyrroline-5-carboxylate synthetase, ATP-grasp domain-containing protein, glutamate decarboxylase, ornithine aminotransferase and aminomethytransferase. Our previous results also indicated that the expression levels of most FAAs metabolism key enzyme genes were significantly changed under the low salinity stress after 7 days treatment. The significantly higher expressions of these genes in salinity adapted individuals compared with control further indicated that these genes contribute to the oyster low salinity adapted response. As previous studies reported, this expression differentiation may be caused by the genetic variation at the regulated regions of these genes between this two groups [51].

In conclusion, our analysis uncovered the taurine, proline, arginine, beta-alanine and glycine metabolic mechanisms under salt stress conditions. The genes expression differentiations between low salinity adapted individuals and controls may further validate the important roles of FAAs for oyster salinity stress adaptation.

### Expanded FAAs metabolic pathways

The Pacific oyster, which thrives in the intertidal zone, must have developed special capabilities to cope with this highly stressful environment [52]. To understand the genomic basis of this oyster’s adaptation to salt stress conditions, we compared gene numbers for aquatic intertidal animals (*C. gigas*, *Capitella capitata*, *Lottia gigantean*, *Strongylocentrotus purpuratus*, *Helobdella robusta*) vs. three stenohaline terrestrial animals (*Drosophila melanogaster*, *Apis mellifera*, *Homo sapiens*) and oysters vs. the seven other species (Table S1).

**Figure 6:**
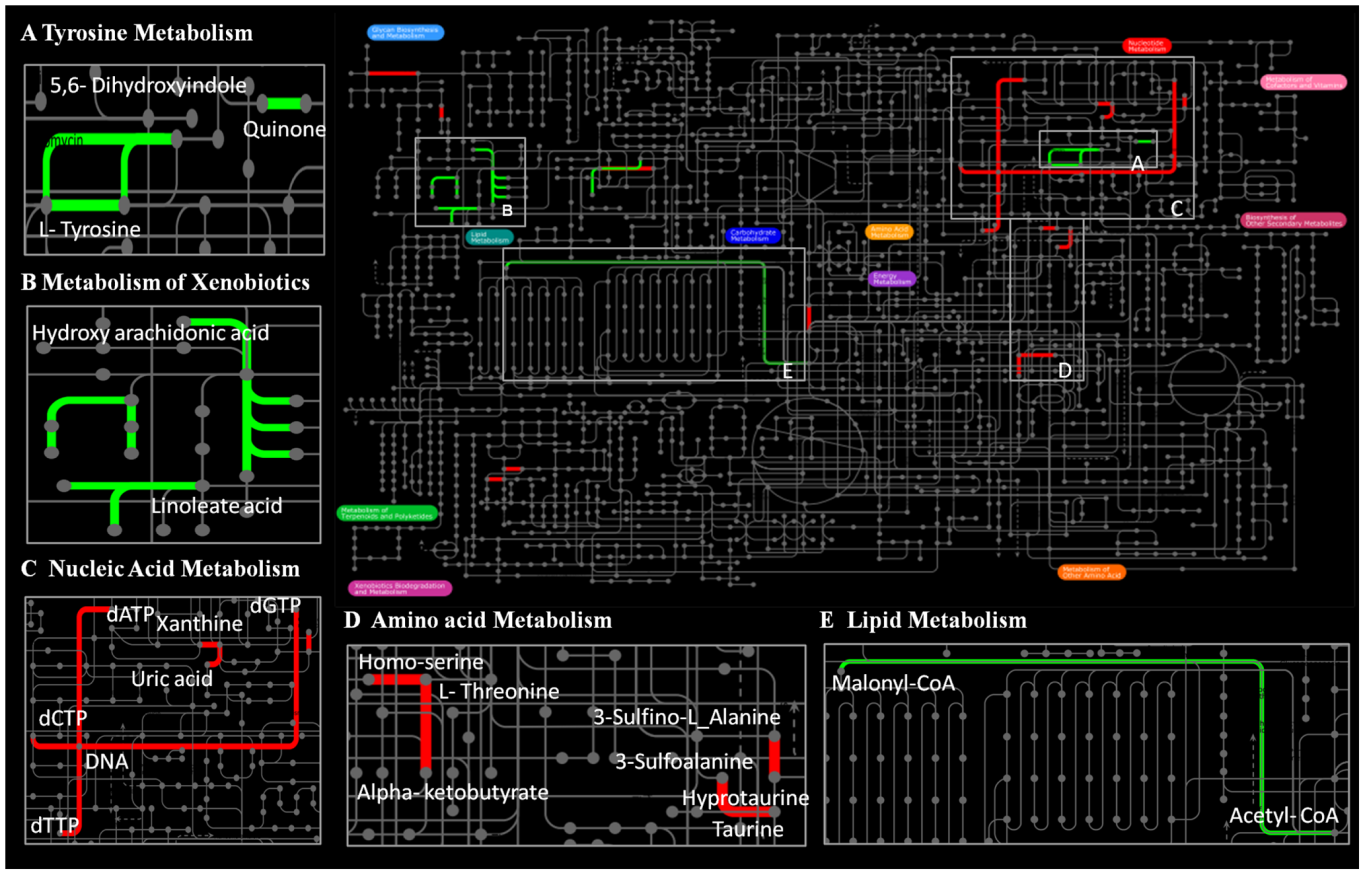
Global metabolic pathway (iPath) analysis for expanding KOs. Green lines represent expanded pathways in intertidal animals (*C. gigas, Capitella capitata, Lottia gigantean, Strongylocentrotus purpuratus and Helobdella robusta*) compared with terrestrial animals (*Drosophila melanogaster, Apis mellifera and Homo sapiens*), and the red line represents the oyster (*C. gigas*) expanded pathways compared with seven other species.

KOs annotations were conducted in eight species genome database (Table S1). We searched for expanded KOs comparing aquatic intertidal animals vs. stenohaline terrestrial animals and oysters vs. the seven other species. The results (Table S5) revealed there were 75 and 52 expanded KOs in aquatic intertidal animals vs. terrestrial animals and oysters vs. the other seven species displayed in Table S1 respectively. iPath is an open-access online tool (http://pathways.embl.de) for visualizing and analyzing metabolic pathways. The interactive viewer provides straightforward navigation through various pathways and enables easy access to the underlying chemicals and enzymes [19]. KOs can be used to map to iPath to investigate their metabolism relationships. To better study the interaction functions of the expanded KOs, we mapped the aquatic intertidal animals expanded KOs and oyster expanded KOs (Table S5) using iPath to investigate their co-expression. The results indicated that the expanded KOs were non-randomly clustered among five metabolic categories (Fig. 6, Table S6). One oyster expanded KOs enriched aspect (red line) displayed in iPath analysis was amino acids metabolism (Fig. 6). Analysis of the maps in this category (Table S6) indicated that several FAAs metabolic pathways were enriched, which included ‘glycine, serine and threonine metabolism’ (map00260), ‘cysteine and methionine metabolism’ (map00270), ‘arginine and proline metabolism’ (map00330), ‘beta-alanine metabolism’ (map00410) and ‘taurine and hypo-taurine metabolism’ (map00430). This indicated that oyster expanded KOs co-expressed in these FAAs metabolism pathways.

We also conducted KEGG maps comparison analysis comparing aquatic intertidal animals vs. three stenohaline terrestrial animals and oysters vs. the seven other species (Table S1). The results indicated that there were 78 and 60 maps expanded in aquatic intertidal animals and oyster respectively (Table S7). Analysis expanded maps, we can see that oyster presented significant greater gene numbers in map00260, 00270, 00410 and 00330 compared with seven other species (Table S1). This result was identified with iPath analysis and indicated that oyster shows a stronger capability regarding glycine, arginine, proline and beta-alanine metabolism. Analysis of the genes counts associated with map00430 ‘taurine and hypotaurine metabolism’ showed that the aquatic intertidal species (including oysters) presented two fold more of these genes than the terrestrial animals, indicating their significant tendency for expansion. This finding may represent one of the most important explanations for the abundance of taurine in oysters. ‘Cysteine and methionine metabolism’ pathway, which was the main resource for taurine synthesis, also expanded in oyster. In conclusion, the map comparison indicated that several FAAs metabolism pathways, which also displayed in iPath, expanded in oyster compared with seven other species.

Overall, the expanded numbers in FAAs metabolism-related pathways may indicate that oysters show a strong capability regarding FAAs metabolism and effectively regulate osmotic pressure to adapt to salt stress. This may be one of the most important mechanisms allowing oysters to adapt to their highly variable salt stress environment.

### Metabolic network involved in salinity adaptation in oysters

**Figure 7:**
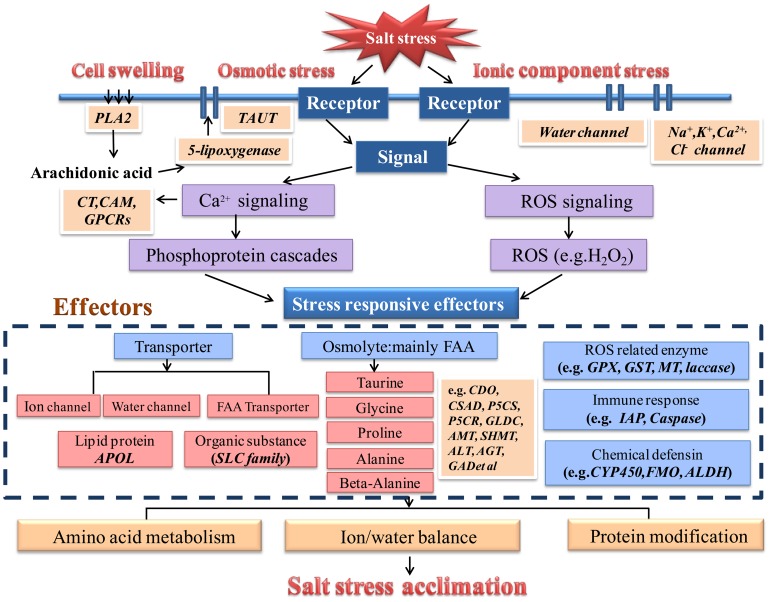
Molecular model of the oyster long-term salt stress response. The map describes salt stress-related signal transduction, effectors and physiological changes in the oyster. The italicized characters in a rectangle with a white border indicate enriched genes under the salt stress response. PLA2: Phospholipase A2, CT: Calcitonin receptor, CAM: Calmodulin-like protein, GPCR: G-protein coupled receptor, TAUT: Taurine transporter, APOL: Apolipoprotein-L, APLP: The amyloid precursor-like protein, CDO: Cysteine dioxygenase, CSAD: Cysteine sulfinic acid decarboxylase, GLDC: Glycine dehydrogenase, AMT: Aminomethytransferase, P5CS: Δ-1- pyrroline-5-carboxylate synthase, P5CR: Δ-1-pyrroline-5-carboxylate reductase, ALT: Alanine transaminase, AGT: Alanine-glyoxylate transaminase, GAD: Glutamate decarboxylase, SHMT: Serine hydroxymethyl transferase, GPX: Glutathione peroxidase, GST: Glutathione S-transferase, MT: Metallothionein, IAP: Inhibitor apoptosis protein, CYP450: Cytochrome P450, FMO: Flavin-containing monooxygenase, ALDH: Aldehyde dehydrogenase.

To investigate the networks involved in oyster salt stress transduction, we further analyses the GO and KEGG (Figure S3) annotation of DEGs. A hypothetical model depicting the components involved in the oyster salt-responsive networks was established (Fig. 7).

First, the oyster perceives osmotic and ionic imbalances through cell membrane receptors. Ion channels (Na^+^, K^+^, Ca^2+^ and Cl^-^ channels) and AQPs were the identified membrane proteins that could receive these signals. Additionally, membrane modification through arachidonic acid metabolism was observed to occur under low salt stress conditions. ‘Arachidonic acid metabolism’ was shown to be enriched in the KEGG pathway analysis under salt stress conditions. In previous studies, it has been revealed that phospholipase A2 (PLA2) mediates the mobilization of arachidonic acid, activated by cell swelling, leading to the release of osmolytes [53]. Our data showed that the transcripts of genes coding for carbonyl reductase, cytochrome P450, arachidonate-5-lipoxygenase, allene oxide synthase-lipoxygenase protein, phospholipase A2 were up- or down-regulated, which may validate this signal transduction pathway in oysters.

Then, salt stress signal transduction pathway was identified. The calcium signaling cascade and phosphorylation regulation were identified through GO analysis based on the term ‘response to stimulus’, which was also found in previous studies by Zhao *et al.* [23]. Many related genes, including the *calcitonin receptor* (*CT*), *calmodulin-like protein*, *G-protein coupled receptor* (*GPCRs*), and *1-phosphatidylinositol-4,5-* *bisphosphate phosphodiesterase epsilon-1*, were observed to be up-regulated. This finding can be interpreted as indicating that salt stress altered membrane fluidity, which induced cytosolic Ca^2+^ oscillations and excited the Ca^2+^ signaling pathway [54]. ROS signal transduction pathways were also identified. The up-regulation of the ROS scavenger *glutathione peroxidase* (*GPX*) under hypo-osmotic stress may support to the idea that GPX catalyzed the reduction of H_2_O_2_, which is mainly induced by low osmotic stress [55].

Third, key salt stress effectors were identified under low osmotic stress. (A) Ion channels (including Na^+^, K^+^, Ca^2+^ and Cl^-^ channels), water channels and osmolyte transporters were activated, which control the ions, water and osmolytes balance. For example, under the ‘transport’ term, the neurotransmitter transporter ‘*sodium- and chloride-dependent glycine transporter*’ was enriched, which played dual roles in the control of neuronal excitability and the osmotic stress response [56]. The enriched lipid transporter ‘*apolipoprotein-L*’ may alter lipid metabolism to maintain energy requirements and the osmotic balance under long-term salt stress [57]. (B) FAAs metabolic pathways (including those for glycine, alanine, beta-alanine, arginine, proline, and taurine) and especially key enzyme genes involved in these pathways (e.g., *CSAD*, *GLD*, *P5CS*, *P5CR*, *AGT*, *ATPGD*) were activated, altering the osmotic status in the oysters and allowing them to adapt to osmotic stress conditions [13]. These pathways were observed to be expanded in intertidal animals compared with stenohaline terrestrial animals, providing one of the most important explanations for oyster adaptation to the intertidal zone. (C) ROS-related genes were enriched. In the analysis of the enriched GO term ‘oxidoreductase’, many genes, including *CYP450, FMO,* and *ALH*, were observed to be enriched, indicating their important roles in substrate oxidation reactions [58]. Previous studies were the first to identify laccase-like activity in the plasma of oysters [59], [60]. In our results, ten copies of the *laccase* genes showed increased expressions, indicating their important roles in the defense against salt stress. Corresponding results have been observed in many plant species [61], [62]. (D) Moreover, many other types of metabolism, including immune responses and lipid metabolism, were shown to be enriched in the KEGG pathway analysis. Regarding the immune responses, *inhibit apoptosis protein* (*IAP*) was observed to be enriched, in accord with previous studies [23]. This protein inhibits apoptosis by binding to the tumor necrosis factor receptor and increases the stress tolerance of animals when it is highly expressed [63]. These various regulatory pathways may exist in oysters to allow them to cope with salt stress. Finally, amino acid metabolism, ion currents and water currents were observed to be balanced to maintain iso-osmotic conditions.

## Conclusions

We displayed the FAAs metabolism pathways for oyster low salinity adaptation using physiological experiments, metabolic pathway expression analyses and genome-wide pathway comparisons. The observed metabolic pathway expansion and up/down-regulation were identified as the most important mechanisms leading to their euryhaline adaptation. FAAs metabolism key enzyme genes were also observed expression differentiations in low salinity adapted individuals compared with control which further confirmed their important roles for oyster low salinity adaptation. The transcriptome data analyses allowed us to describe the salt stress signal transduction network in oysters, which was basically consistent with the changes seen in other mollusks. The genes mentioned in this article displayed in Table S8.

## Supporting Information

Figure S1 **Multi-alignment of CSAD between different species.** The following species were included: *Mus musculus* CASD (NP_659191), *Takifugu rubripes* CSAD (ABF2245), *Homo sapiens* CSAD (NP_057073), *Cyprinus carpio* CSAD (BAE73113), and *Danio rerio* CSAD (NP_001007349). A rectangle indicates the ‘ZnF_GATA’ domain.
(DOCX)

Figure S2 **Expression levels of detected 17 FAAs metabolism key enzyme genes in two salinity adapted groups.**
(DOCX)

Figure S3 **The enriched pathways involving salt stress-responsive genes.** The pathways were identified by KEGG mapping using the chi-square test (*P*<0.05). The x-axis values indicate the -log_10_ (*P*-value) of the enriched KEGG maps.
(DOCX)

Table S1 **Genome Data resources used in global metabolic pathways (iPath) analysis.**
(XLSX)

Table S2 **Sequences of the primers used in this study for quantitative real-time PCR analysis.**
(XLSX)

Table S3 **Marker genes for the various salinities treatments.**
(XLSX)

Table S4 **Ion channel and water channel genes expressions under different salinities.**
(DOCX)

Table S5 **Expanded KOs between different species.**
(XLSX)

Table S6 **Co-expanded pathways in the iPath map.**
(XLSX)

Table S7 **Expanded maps between different species.**
(XLSX)

Table S8 **Gene IDs included in this manuscript.**
(XLSX)
