# Supplementary material for: Genome and Transcriptome Analyses Provide Insight into the Euryhaline Adaptation Mechanism of Crassostrea gigas
Source: PLoS One. 2013 Mar 12;8(3):e58563. doi: 10.1371/journal.pone.0058563 (PMC3595286; doi:10.1371/journal.pone.0058563)
Supplement: Figure S1 — Multi-alignment of CSAD between different species. The following species were included: Mus musculus CASD (NP_659191), Takifugu rubripes CSAD (ABF2245), Homo sapiens CSAD (NP_057073), Cyprinus carpio CSAD (BAE73113), and Danio rerio CSAD (NP_001007349). A rectangle indicates the ‘ZnF_GATA’ domain. (DOCX) [file pone.0058563.s001.docx]

**FigureS2. Multi-alignment of CSAD between different species.** The species include: *Mus musculus* CASD (NP_659191), *Takifugu rubripes* CSAD (ABF2245), *Homo sapiens* CSAD (NP_057073), *Cyprinus carpio* CSAD (BAE73113), *Danio rerio* CSAD (NP_001007349). The sequence with rectangle indicated the ‘ZnF_GATA’ domain.
