# Supplementary material for: Genome and Transcriptome Analyses Provide Insight into the Euryhaline Adaptation Mechanism of Crassostrea gigas
Source: PLoS One. 2013 Mar 12;8(3):e58563. doi: 10.1371/journal.pone.0058563 (PMC3595286; doi:10.1371/journal.pone.0058563)
Supplement: Figure S2 — Expression levels of detected 17 FAAs metabolism key enzyme genes in two salinity adapted groups. (DOCX) [file pone.0058563.s002.docx]

A

B

**Fig. S2: Expression levels of detected 17 FAAs metabolism key enzyme genes in two salinity adapted groups. (A)** The 17 genes include: aminomethytransferase, glycine dehydrogenase, glycine hydroxymethyltransferase, Δ-1-pyrroline-5-carboxylate synthase, Δ 1 -pyrroline-5-carboxylate reductase, ornithine aminotransferase, arginase, alanine transaminase, alanine-glyoxylate transaminase, glutamate decarboxylase (two copies), spermidine synthase, aldehyde dehydrogenase, ATP-grasp domain-containing protein, cysteine sulfinic acid decarboxylase (two copies), taurine transporter. **(B)** The significant changed genes: P5CS means: Delta-1-pyrroline-5-carboxylate synthetase; ATPGD1 means ATP-grasp domain-containing protein; GAD means glutamate decarboxylase; OAT means ornithine aminotransferase; AMT means aminomethytransferase. Statistical analysis was performed by independent samples T test, ***P*<0.01.
