# Supplementary material for: Genome and Transcriptome Analyses Provide Insight into the Euryhaline Adaptation Mechanism of Crassostrea gigas
Source: PLoS One. 2013 Mar 12;8(3):e58563. doi: 10.1371/journal.pone.0058563 (PMC3595286; doi:10.1371/journal.pone.0058563)
Supplement: Figure S3 — The enriched pathways involving salt stress-responsive genes. The pathways were identified by KEGG mapping using the chi-square test (P<0.05). The x-axis values indicate the -log10 (P-value) of the enriched KEGG maps. (DOCX) [file pone.0058563.s003.docx]

**A**

**FigureS4. The enriched pathways of salt stress-responsive genes.** The pathways were identified by KEGG mapping. Enrichment analysis for a given gene list was carried out using chi-square test (*P*<0.05). The x axis values indicated the –log_10_(P-value) of the enriched KEGG maps.
