# Supplementary material for: Genome and Transcriptome Analyses Provide Insight into the Euryhaline Adaptation Mechanism of Crassostrea gigas
Source: PLoS One. 2013 Mar 12;8(3):e58563. doi: 10.1371/journal.pone.0058563 (PMC3595286; doi:10.1371/journal.pone.0058563)
Supplement: Table S4 — Ion channel and water channel genes expressions under different salinities. (DOCX) [file pone.0058563.s007.docx]

|  | | **mRNA expression level (RPKM)** | | | | | | |
| --- | --- | --- | --- | --- | --- | --- | --- | --- |
| **Name** | **Gene ID** | **salt5** | **salt10** | **salt15** | **salt20** | **salt25** | **salt30** | **salt40** |
| NaV | CGI_10003777 | 29.07 | 20.94 | 17.97 | 33.06 | 40.57 | 50.73 | 39.98 |
|  | CGI_10026710 | 11.10 | 8.41 | 7.66 | 13.57 | 21.64 | 15.07 | 13.81 |
| KV | CGI_10015111 | 11.28 | 6.19 | 9.54 | 15.51 | 15.62 | 18.01 | 12.42 |
|  | CGI_10022859 | 4.88 | 2.60 | 2.36 | 3.90 | 4.66 | 6.46 | 3.60 |
|  | CGI_10024636 | 15.32 | 7.66 | 5.10 | 13.23 | 11.93 | 12.11 | 12.97 |
|  | CGI_10027006 | 6.80 | 3.72 | 4.26 | 11.01 | 7.56 | 12.47 | 7.06 |
| BKCa | CGI_10011873 | 8.88 | 21.93 | 13.27 | 7.31 | 12.54 | 9.59 | 8.84 |
| CaC | CGI_10013580 | 7.73 | 12.65 | 10.79 | 5.46 | 4.734 | 5.08 | 6.71 |
| ClC | CGI_10012229 | 17.78 | 15.03 | 14.57 | 7.15 | 10.13 | 7.38 | 8.04 |
| AQP | CGI_10024446 | 3.79 | 0.61 | 0.30 | 5.65 | 6.80 | 10.99 | 7.19 |
|  | CGI_10013723 | 28.08 | 4.19 | 3.35 | 38.49 | 46.39 | 50.59 | 18.05 |
|  | CGI_10001489 | 5.81 | 2.37 | 1.45 | 12.21 | 5.48 | 11.26 | 5.35 |

**Table S4:** Ion channel and water channel genes expressions under different salinities.

NaV means voltage-gated Na^+^ channel; KV means voltage-gated K^+^ channel; BKCa means calcium-activated K^+^ channel; CaC means Ca^2+^ channel; ClC means Cl^-^ channel; AQP means aquaporins. Salt 40 was high salinity, salt 30 was the normal salinity, salt 5, 10, 15, 20 and 25 were the low salinity. As previous reported, salt 5 was special and may exceed oyster salinity tolerance ranges [18].
